# Stripped: contribution of cyanobacterial extracellular polymeric substances to the adsorption of rare earth elements from aqueous solutions

**DOI:** 10.3389/fbioe.2023.1299349

**Published:** 2023-12-20

**Authors:** Michael Paper, Patrick Jung, Max Koch, Michael Lakatos, Tom Nilges, Thomas B. Brück

**Affiliations:** ^1^ Werner Siemens-Chair of Synthetic Biotechnology, Department of Chemistry, School of Natural Sciences, Technical University of Munich, Garching, Germany; ^2^ Integrative Biotechnology, University of Applied Sciences Kaiserslautern, Pirmasens, Germany; ^3^ Synthesis and Characterization of Innovative Materials, Department of Chemistry, School of Natural Sciences, Technical University of Munich, Garching, Germany; ^4^ Department of Aerospace and Geodesy, TUM AlgaeTec Center, Ludwig Bölkow Campus, Taufkirchen, Germany

**Keywords:** extracellular polymeric substances, polysaccharides, *Komarekiella*, *Nostoc*, *Desmonostoc*, biosorption

## Abstract

The transformation of modern industries towards enhanced sustainability is facilitated by green technologies that rely extensively on rare earth elements (REEs) such as cerium (Ce), neodymium (Nd), terbium (Tb), and lanthanum (La). The occurrence of productive mining sites, e.g., is limited, and production is often costly and environmentally harmful. As a consequence of increased utilization, REEs enter our ecosystem as industrial process water or wastewater and become highly diluted. Once diluted, they can hardly be recovered by conventional techniques, but using cyanobacterial biomass in a biosorption-based process is a promising eco-friendly approach. Cyanobacteria can produce extracellular polymeric substances (EPS) that show high affinity to metal cations. However, the adsorption of REEs by EPS has not been part of extensive research. Thus, we evaluated the role of EPS in the biosorption of Ce, Nd, Tb, and La for three terrestrial, heterocystous cyanobacterial strains. We cultivated them under N-limited and non-limited conditions and extracted their EPS for compositional analyses. Subsequently, we investigated the metal uptake of a) the extracted EPS, b) the biomass extracted from EPS, and c) the intact biomass with EPS by comparing the amount of sorbed REEs. Maximum adsorption capacities for the tested REEs of extracted EPS were 123.9–138.2 mg g^−1^ for *Komarekiella* sp. 89.12, 133.1–137.4 mg g^−1^ for *Desmonostoc muscorum* 90.03, and 103.5–129.3 mg g^−1^ for Nostoc sp. 20.02. A comparison of extracted biomass with intact biomass showed that 16% (*Komarekiella* sp. 89.12), 28% (*Desmonostoc muscorum* 90.03), and 41% (*Nostoc* sp. 20.02) of REE adsorption was due to the biosorption of the extracellular EPS. The glucose- rich EPS (15%–43% relative concentration) of all three strains grown under nitrogen-limited conditions showed significantly higher biosorption rates for all REEs. We also found a significantly higher maximum adsorption capacity of all REEs for the extracted EPS compared to cells without EPS and untreated biomass, highlighting the important role of the EPS as a binding site for REEs in the biosorption process. EPS from cyanobacteria could thus be used as efficient biosorbents in future applications for REE recycling, e.g., industrial process water and wastewater streams.

## 1 Introduction

Rare earth elements (REEs), such as cerium (Ce), neodymium (Nd), terbium (Tb), and lanthanum (La), are expensive metals used in various modern electronic devices, like mobile phones, computers, and LCDs, and in emerging green technologies, e.g., in wind turbines or electric cars. Beyond their incorporation into these electronic devices and their pivotal role in manufacturing processes (Klingelhöfer et al., 2022), REEs also find extensive applications as medical products or as fertilizers for crops in agriculture. In addition, they are of new interest to veterinary practice since antibiotics as feed additives that promote growth have been banned due to global health concerns. Furthermore, REEs have been shown to have various beneficial impacts on animal growth and performance, leading to a discussion because this comes with adverse ecotoxicological effects (Abdelnour et al., 2019).

As such, there is a rising global demand for REEs that has become eminent, especially in recent years, resulting in a pronounced increase in production. This surge in REE output has led to the release of substantial quantities of anthropogenic REEs into rivers and streams through wastewater discharges. During a 15-year monitoring approach, e.g., it could be estimated that the Gironde Estuary in the Bordeaux Metropole of France transports more than 27 kg per year of the REE gadolinium (Gd), which is used as a contrast agent for magnetic resonance imaging (MRI) (Lerat-Hardy et al., 2019). Unfortunately, this and other studies also showed that wastewater treatment plants (WWTPs) often exhibit limited efficiency in removing REEs. Consequently, these valuable components are highly diluted in wastewater streams and rivers, where efficient and environmentally friendly recovery methods are hard to apply (Pereao et al., 2018). Several procedures, like precipitation, ion exchange, electrochemical methods, reverse osmosis, and adsorber resins, are utilized, but they come with high process costs and environmental impact due to toxic resins or inefficient recovery of highly diluted REEs (Chen et al., 2018; Jyothi et al., 2020; Pramanik et al., 2020). According to a study published in 2023, the recovery of some metals from highly diluted sources, e.g., from industrial wastewater, will become an economically viable option in the years to come, especially as the demand for such metals keeps rising (DuChanois et al., 2023). The elements regarded as high-priority metals are energy-intensive to obtain, geologically rare, and essential for high-tech sectors. In this context, REEs were listed alongside other metals like gallium, vanadium, or lithium.

In contrast to traditional recycling processes, microbiological and biotechnological approaches have gained increasing attention recently as they are considered sustainable and associated with low costs (da Costa et al., 2020; Dev et al., 2020). Among these processes, biosorption has been studied, a process describing the passive uptake of substances, such as metal ions, by biological materials, including biomass derived from diverse microorganisms (Giese, 2020; Brown et al., 2023). In the context of bioremediation, biosorption has been successfully applied to effectively sequester heavy metals from diluted solutions (Cho and Kim, 2003; Gupta et al., 2000). Along with heterotrophic microorganisms like *Escherichia coli* (e.g., Park et al., 2020) or *Saccharomyces cerevisiae* (Ojima et al., 2019), algae and cyanobacteria have emerged as particularly promising candidates for such purposes (e.g., Romera et al., 2006; Fischer et al., 2019; Al-Amin et al., 2021; Paper et al., 2023).

Cyanobacteria are assumed to have evolved approximately 3.5 billion years ago (Sánchez-Baracaldo and Cardona, 2020) and are adapted to nearly every habitat on Earth, from the aquatic systems of oceans and freshwater rivers to hot and cold deserts of extreme environments (e.g., Makhalanyane et al., 2015). Moreover, cyanobacteria have established stable symbiotic relationships between fungi, bryophytes, ferns, and plants (e.g., Rai et al., 2002). Fischer et al. (2019) showed that two heterocystous (nitrogen-fixing) cyanobacterial strains of the genus *Anabaena* were able to promote the enrichment of REE europium, samarium, and neodymium, while other species, such as *Arthrospira platensis*—which is already used in industrial food-additive production worldwide—can act as a safe and efficient bioremediator of erbium (Er)-contaminated wastewater (Yushin et al., 2022).

However, several mechanisms, such as ion exchange, complexation, or electrostatic attraction, can be simultaneously involved in the process of metal adsorption (Bilal et al., 2018). The contribution of EPS to the overall adsorption of REEs in cyanobacterial biomass has not been part of extensive research yet. In this context, the following major factors have been discussed for cyanobacteria: 1) the chemical composition of extracellular polymeric substances (EPS), also known as sheaths, capsules, and mucilage built by cyanobacteria (De Philippis et al., 2011), 2) the characteristics of their outer cell wall (Singh et al., 1998), 3) the viability state of their biomass (Singh, 2020), and 4) intracellular active uptake (bioaccumulation) vs. extracellular passive binding (biosorption, Olguín and Sánchez-Galván, 2012).

Following a contradicting discussion, biosorption is likely influenced by all of these factors, including the type of the metal ion itself, and thus metal adsorption is specific for each cyanobacterial biomass and each metal being taken up (Wilke et al., 2006; Dixit and Singh, 2013; Rzymski et al., 2014). Some studies, e.g., have presented evidence that REEs are adsorbed by living biomass and subsequently transported into the cell lumen and, at the same time, excluded the role of extracellular polymeric substances (EPS) (Fischer et al., 2019). It has also been reported that the viability of the biomass appears to be the most important factor in the process (Acharya et al., 2012). In contrast, others demonstrated that solely specific compounds of EPS, e.g., the large molecular weight and sulfated polysaccharide sacran from *Aphanothece sacrum*, are responsible for the biosorption of the REE Nd (Okajima et al., 2010).

Hence, EPS must play a major role in biosorption when considering that most cyanobacteria produce these substances in copious amounts as they alleviate environmental stress conditions, prevent desiccation, store UV-protecting pigments such as scytonemin, and act as temporary storage to balance the intracellular C:N ratio (Tamaru et al., 2005; Pannard et al., 2016). As such, two forms of EPS can be distinguished: the free fraction composed of soluble extracellular polymeric substances (S-EPS; Underwood et al., 1995) and the particulate fraction corresponding to the transparent exopolymer particles known from, e.g., cyanobacterial blooms in water bodies (TEP; Passow and Alldredge, 1995). An overproduction of both types of EPS is triggered under nutrient-limited (N or P) conditions (Myklestad, 1995; Reynolds, 2007; Qian et al., 2022), during which photosynthetic carbon can still be accumulated, while protein production and growth are inhibited (De Philippis and Vincenzini, 1998). Consequently, carbon overflow can either be stored as reserve compound or excreted as EPS, which becomes especially apparent in terrestrial cyanobacterial species, such as *Nostoc commune*, that forms macroscopic thalli, which considerably improves the water holding capacity (Tamura et al., 2005). S-EPS have been reported to exhibit higher metal adsorption capacities due to their particularly high number of carboxyl and hydroxyl groups (Pereira et al., 2011; Mota et al., 2016).

Compared to EPS produced by other microorganisms, cyanobacterial EPS exhibit several unique compositional features, such as the presence of uronic acids or a high content of sulfate groups (Klock et al., 2007; Pereira et al., 2009). The accumulation of functional groups that are typically found in cyanobacterial EPS leads to particular anionic properties, which are beneficial for the attraction of positively charged metal ions.

Furthermore, during the biosorption process, functional units present in EPS, such as certain proteins, which can form complexes with metal cations, have frequently been discussed as the binding site (De Philippis et al., 2011). If the EPS should not be involved in the biosorption process, the REE must penetrate the EPS, which can be difficult because, in many species, these substances create a multi-layered sheath, and its thickness can reach up to double the diameter of the actual cell size (Mota et al., 2022).

In order to step into this ongoing discussion, we evaluated the role of EPS in biosorption of cerium (Ce), neodymium (Nd), terbium (Tb), and lanthanum (La) from three terrestrial, heterocystous cyanobacterial strains: *Nostoc* sp. 20.02 (epiphyte on lichen *Peltigera* sp.), *Desmonostoc muscorum* 90.03/PCC7903 (isolated from soil), and *Komarekiella* sp. 89.12 (hypoliths on quartz), which have been reported to be efficient REE adsorbers in our previous studies (Paper et al., 2023). We cultivated all three strains under N-limited and non-limited conditions, forcing EPS production, and harvested the EPS for structural and compositional analyses. Subsequently, we incubated 1) the separated EPS, 2) the EPS-free biomass, and 3) the intact biomass with EPS with Ce, Nd, Tb, and La, respectively. Afterward, we evaluated and compared the amount of each sorbed REE.

## 2 Materials and methods

### 2.1 Cultivation of cyanobacterial strains

Three terrestrial and nitrogen-fixing cyanobacterial strains, *Nostoc* sp. 20.02 (epiphyte on lichen *Peltigera* sp.; Germany), *Desmonostoc muscorum* 90.03/PCC7903 (soil, United States), and *Komarekiella* sp. 89.12 (hypolithic on quartz, Namibia, South Africa) were used for the study. Their phylogenetic placement based on their complete 16S rRNA sequence was recently elucidated, from which it can be deduced that *Komarekiella* sp. 89.12 represents a novel species (Paper et al., 2023).

All strains were inoculated with approximately 0.1–0.3 g of wet biomass from a stock culture (17°C; light/dark rhythm 16:8 h; 30 μmol photons m^−2^ s^−1^) in 1-L bubble columns containing BG11 cultivation medium and additionally in nitrogen-depleted BG 11_0_ medium (Lama et al., 1996). In the bubble columns, they were cultivated at 23°C under a light/dark rhythm of 16:8 h at 300 μmol photons m^−2^ s^−1^ photosynthetic photon flux density for 4 weeks. All cultivated cells were first harvested using two sieves of 0.5 mm and 0.1 mm and then using paper filters of 40 µm openings. Afterward, wet biomass was dried by lyophilization.

In addition, all strains were cultivated on 0.9% solidified BG11 medium for 4 weeks in order to visualize their EPS structures by light microscopy under the conditions described above.

### 2.2 Microscopy

All three strains were visualized through differential interference contrast (DIC) microscopy using an Olympus BX51 microscope (Evident Europe GmbH, Hamburg, Germany) equipped with ×10, ×20, ×40, and ×100 magnifications, oil immersion equipped with a MicroLive multi-format camera (Lifesolution, Bremen, Germany), and software MicroLive (v5.0). To visualize the sheath material of the cells, ACN staining was performed (20:1:1 mix; 0.1 g Astra blue in 79.5 mL H_2_O and 2.5 mL acetic acid; 0.1 g chrysoidine in 100 mL H_2_O; and 0.1 g new fuchsine in 100 mL H_2_O; Carl Roth, Karlsruhe, Germany), which allows differentiation of structures according to colors due to the binding characteristics of the substances. Acid mucopolysaccharides are stained blue by Astra blue, cellulose and lignin are stained red by new fuchsine, and hydrophobic substances, such as cutin, are stained yellow.

### 2.3 Separation of EPS

Wet biomass was harvested as described above and used for EPS extraction (modified after Strieth et al., 2020). The wet biomass was collected in 50-mL tubes, and five times the amount (v/w) of ddH_2_O preheated to 55°C was added. The tubes were then mixed for 60 min in an overhead shaker at 55°C in an incubator, followed by ultrasonic treatment for 5 min (at 240 W and 40 kHz, Emmi-H60, EMAG, Mörfelden, Germany) and centrifugation at 14,000 rcf. The supernatant was transferred into 50-mL centrifuge tubes for lyophilization (at 0.1 mbar and 21°C, Epsilon 2-4 LSCplus, Christ, Osterode, Germany).

### 2.4 Metal adsorption experiments

Metal adsorption was tested for EPS, untreated biomass, and biomass after EPS separation. The experiments were carried out following an experimental setup described by Heilmann et al. (2015) and Heilmann et al. (2021). Single-metal REE solutions with a concentration of 10 mM and a starting pH value of 5 ± 0.2 were used to determine the maximal adsorption capacity of the tested biomasses. The influence of the pH value on metal biosorption and experiments on adsorption kinetic carried out by varying the incubation time of the biomass in cerium(III) nitrate solutions were determined in previous studies on the untreated biomass (see Paper et al., 2023). Therefore, to compare the untreated and treated conditions investigated here, an optimal pH value of 5 was assumed to be efficient for a preliminary standard protocol. To determine the maximum adsorption capacity (*Q*), 10–20 mg of dry biomass was weighed into centrifuge tubes and incubated in 2 mL of metal solutions for 3 h under constant shaking at room temperature. Afterward, the metal adsorption to the tested biomass was calculated by dividing the changes in metal concentration by the amount of incubated biomass (see Eq. 1).

$$\;Q=\frac{n_{i}-\;n_{f}\;}{m}=\frac{\left(c_{i}-c_{f}\right)\times V}{m},$$ (1)

where *Q* = adsorption capacity, *n*_*i*_ = initial amount of substance, *n*_*f*_ = final amount of substance after incubation, *c*_*i*_ = initial metal concentration, *c*_*f*_ = final metal concentration after incubation, *V* = volume, and *m* = weight of biomass.

### 2.5 FT-IR analysis of cyanobacterial biomass and EPS

Infrared (IR) spectroscopy is a valuable method for determining the qualitative composition of organic functional groups. In this study, it was used to detect and identify interactions of metal cations with functional groups in isolated EPS samples. Following incubation in cerium(III) nitrate solution (1 µmol 1 mg^−1^ biomass) for 2 h, the samples were lyophilized. A FT-IR spectrometer (Nicolet iS50R, Thermo Fisher Scientific, Waltham, US) equipped with an attenuated total reflection multi-range diamond sampling station (iS50 ATR) was used to obtain the IR spectra. The IR spectra were recorded in a range from 400 to 4,000 cm^−1^ for each sample.

### 2.6 HPLC analysis of EPS

Polysaccharides are known to play an important role in biosorption processes due to their ability to form complexes with metal cations (Joly et al., 2020). As EPS comprise chemically complex polymeric carbohydrates, the monomeric sugar composition of the extracted EPS samples was analyzed using an HPLC-based method. The monomeric sugar composition of the cyanobacterial biomass was determined for all three selected strains following a reported protocol (Jurkowski et al., 2022). Each sample was hydrolyzed with 2% H_2_SO_4_ in an autoclave for 1 h at 121°C at 1 bar in order to release monomeric carbohydrate building blocks from the samples constituting polymeric carbohydrates. Afterward, each sample was centrifuged at 10,000 rcf for 10 min. Following hydrolysis, the solutions were neutralized with calcium carbonate (pH 7). Precipitated calcium salt was removed by centrifugation at 10,000 rcf for 10 min after neutralization. The supernatant was frozen at −20°C overnight. Subsequently, the samples were heated to 5°C and centrifuged at 10,000 rcf for 10 min to remove any residual precipitate. Sugar analysis was carried out using an HPLC system (Agilent Infinity II LC 1260, Agilent Technologies, Waldbronn, Germany) equipped with an autosampler, quaternary pump, column oven, DAD, and a Shodex RI detector (Showa Denko Europe GmbH, Munich, Germany). Prior to injection, each sample was filtered using modified PES 500-µL centrifugal filters (VWR, Ismaning, Germany) with a cut-off of 10 kDa. In a subsequent step, the monomeric sugar mixture resulting from chemical hydrolysis was analyzed using the HPLC system previously described. A Rezex ROA-Organic Acid H+ (8%) ion-exclusion column (300 mm, 7.8 mm internal diameter; Phenomenex Ltd., Aschaffenburg, Germany) was used for the isocratic separation with 5 mM sulfuric acid at a flow rate of 0.5 mL min^−1^ and a temperature of 70°C.

### 2.7 Statistical analyses

Analyses were performed using the software package PAST (Hammer et al., 2001). Multivariate normality is assumed by a number of multivariate tests. PAST computes Mardia’s multivariate skewness and kurtosis with tests based on chi-squared (skewness) and normal (kurtosis) distributions. Differences in the adsorption capacity of the tested biomass samples were statistically evaluated with a two-sided *t*-test and one-way ANOVA followed by a *post hoc* analysis according to the Scheffé test (N-limited condition) or by one-way and two-way ANOVA tests (multivariate treatment conditions) depending on the selected dependent and independent variables and verified assuming equal variances by Tukey’s *post hoc* test regarding different factors and interactions (comparison of adsorption capacities).

## 3 Results

### 3.1 Composition of extracellular polymeric substances

**FIGURE 1:**
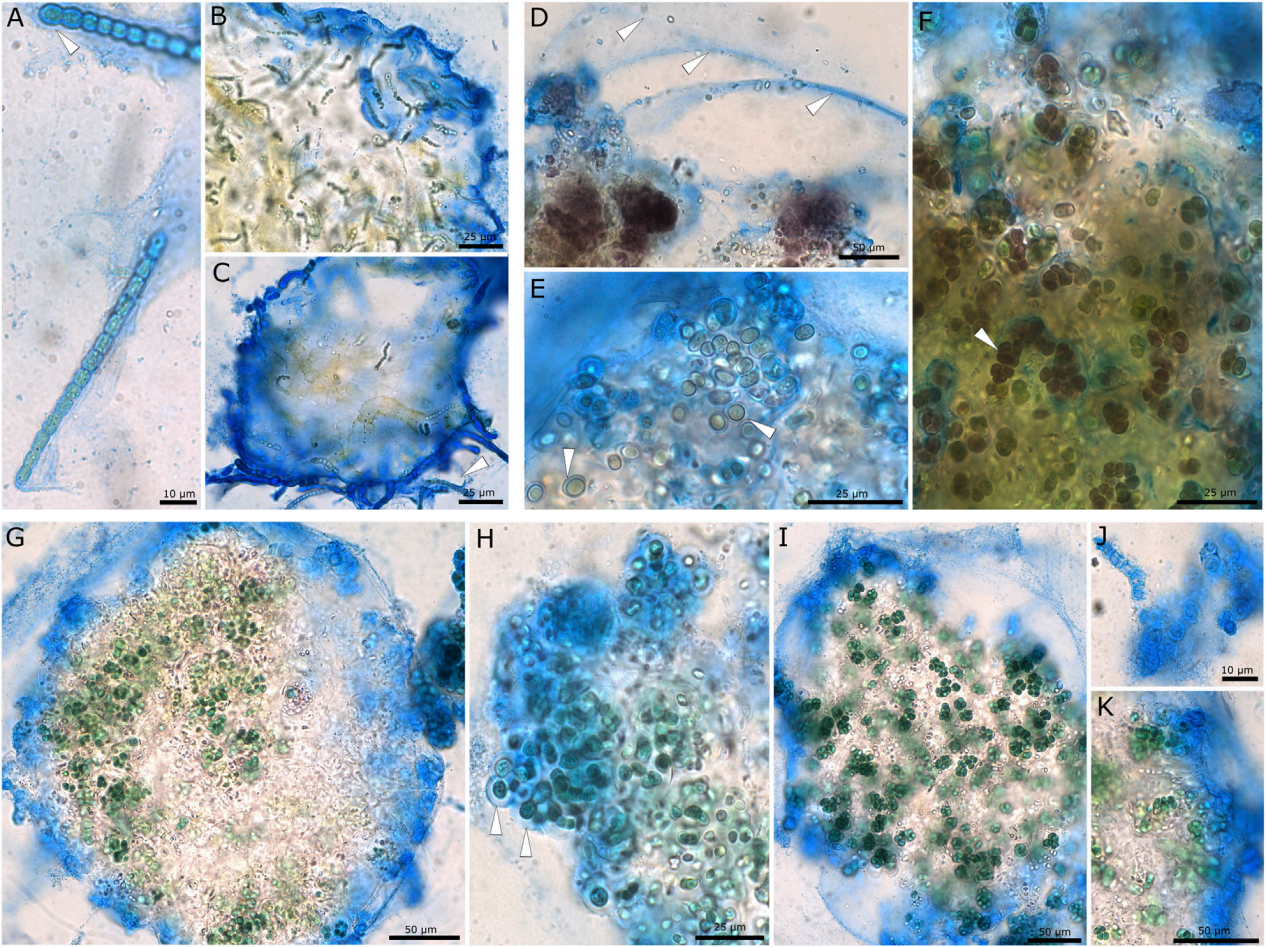
Microscopic images showing EPS of cyanobacterial strains stained with ACN solution. **(A–C)** *Desmonostoc muscorum* 90.03 with thin EPS on hormogonia **(A,C)** and a limited, firm, and wide common sheath surrounding micro-colonies **(B,C)**. **(D–F)** Brownish pigmented cells of *Nostoc* sp. 20.02. **(D)** Wide, diffluent, and hardly limited EPS (arrows), **(E)** akinetes with firm sheaths, and **(F)** small cell packages and multiseriate filaments (arrow) encapsulated by wide, limited sheaths. **(G–K)** *Komarekiella* sp. 89.12 with a limited common sheath encapsulating micro-colonies **(G)**, wide, non-lamellated sheaths surrounding single cells (arrows) **(H)**, small cell packages surrounded by a common sheath **(I)**, firm and narrow capsules of dead cells **(J)** and layers of outer EPS membranes **(K)**.

EPS staining with ACN solution (Figure 1) showed that the structure of the three investigated strains differed. *Desmonostoc muscorum* 90.03 formed thin and firm EPS sheaths around hormogonia (Figures 1A–C arrows), while adult cells loosely grouped in micro-colonies surrounded by a common, limited, and firm sheath. The strain *Nostoc* sp. 20.02 showed the greatest amplitude of cell differentiation and life stages ranging from adult micro-colonies (Figure 1D) to akinetes (Figure 1E) and multiseriate filaments (Figure 1F). During most stages, cells were brownish and surrounded by excess EPS material. Adult micro-colonies were surrounded by a very wide, diffluent, hardly limited, soft, and multi-layered EPS sheath (Figure 1D, arrows). At the same time, akinetes and filaments were encapsulated in firm and limited sheaths (Figures 1E, F). *Komarekiella* sp. 89.12 mainly formed small, densely packed cell packages that were grouped within shared EPS material (Figures 1G, I). The micro-colonies were surrounded by a common, limited, and more or less narrow sheath, while the single cells and small cell packages were coated by wide, limited, and non-layered EPS (Figure 1H, arrows) or firm, narrow, and hard capsules (Figures 1J, K).

**FIGURE 2:**
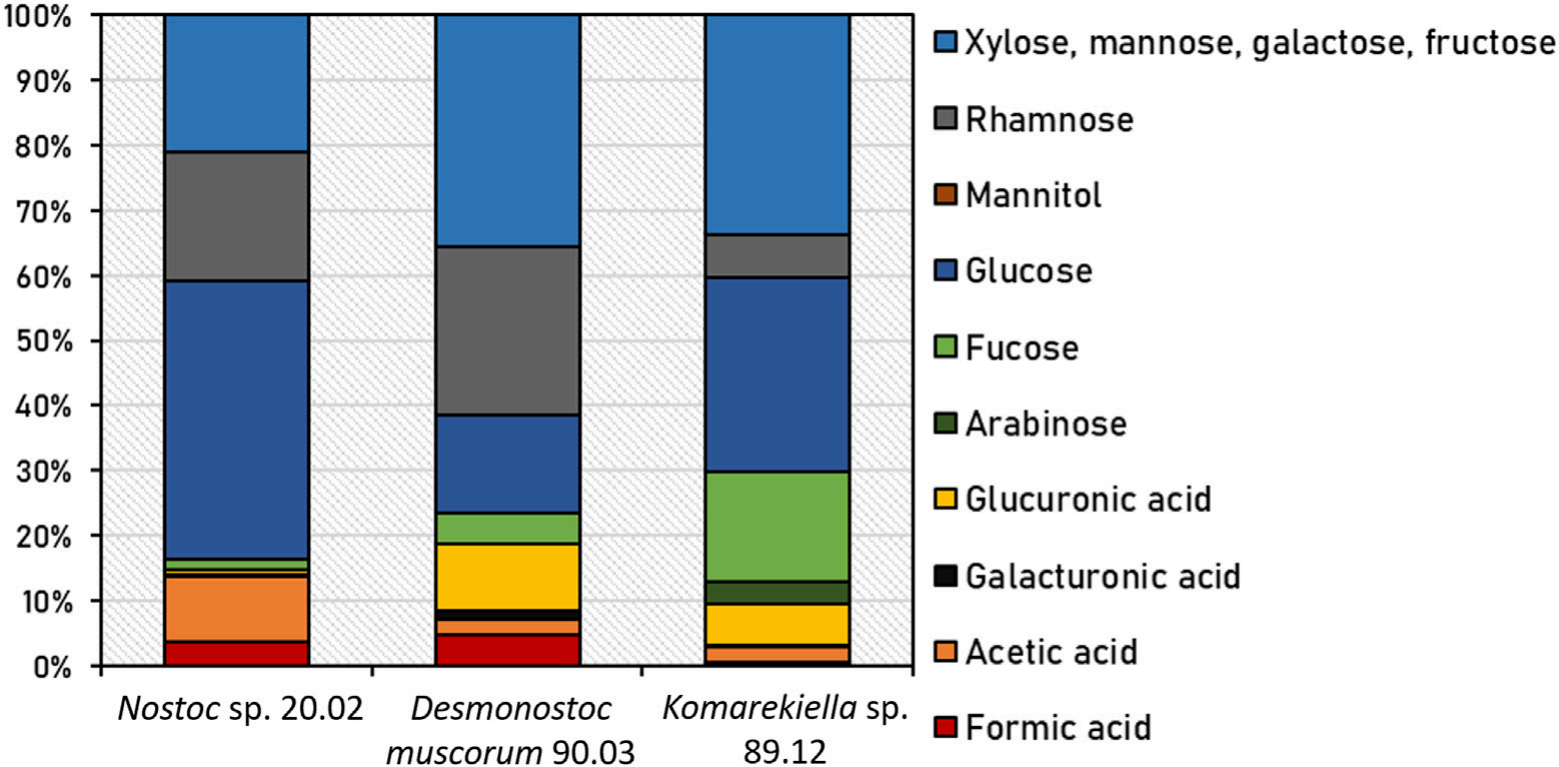
Relative content of detected organic acids and sugar monomers of EPS from *Nostoc* sp. 20.02, *Desmonostoc muscorum* 90.03, and *Komarekiella* sp. 89.12 after chemical hydrolysis with 2% H_2_SO_4_. Underlying data are listed in Supplementary Table S1.

Following chemical hydrolysis with 2% H_2_SO_4_, the monomeric sugar composition of isolated EPS samples from *Nostoc* sp. 20.02, *Desmonostoc muscorum* 90.03, and *Komarekiella* sp. 89.12 was determined using HPLC analysis (Figure 2; Supplementary Table S1). All strains differed in their relative content of detected organic acids and sugar monomers of EPS. The main components of EPS from *Nostoc* sp. 20.02 were 42.7% glucose, 20.0% rhamnose, and 21.0% xylose, mannose, galactose, or fructose (differentiation was not possible because of peak-overlapping). EPS of *Desmonostoc muscorum* 90.03 comprised 36% xylose, mannose, galactose, or fructose, 26.0% rhamnose, and 15.0% glucose. With 10.3%, the portion of glucuronic acid was relatively high. The EPS isolated from *Komarekiella* sp. 89.12 also had high portions of glucose at 29.8% and those of xylose, mannose, galactose, or fructose at 33.7%. Compared to the other samples, the EPS of *Komarekiella* sp. 89.12 included relatively high amounts of fucose monomers at 16.9%.

### 3.2 Maximum adsorption capacity of EPS for REEs under nitrogen-limited vs. non-limited conditions

**FIGURE 3:**
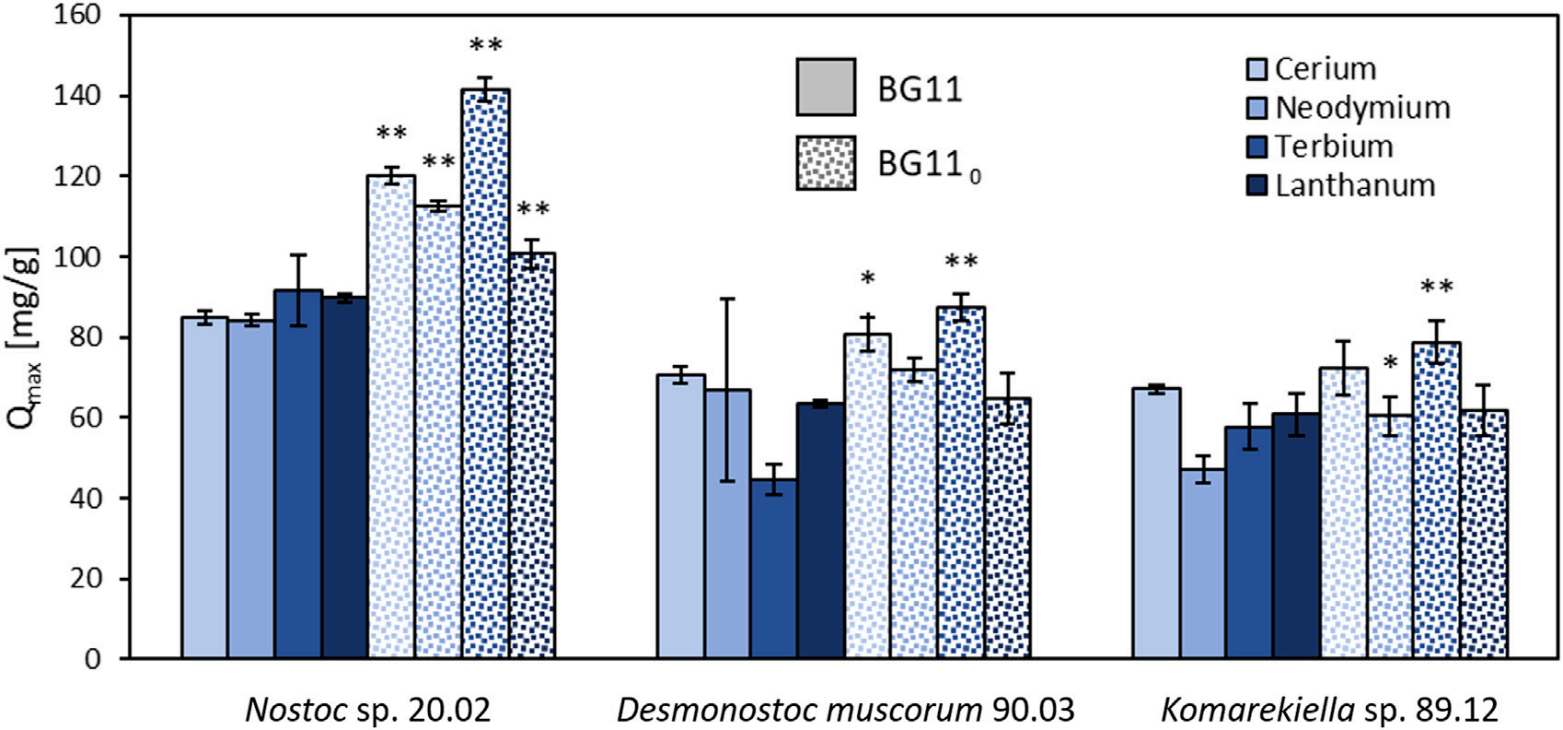
Maximum adsorption capacity for cerium, neodymium, terbium, and lanthanum of the lyophilized biomass (including EPS) of *Nostoc* sp. 20.02, *Desmonostoc muscorum* 90.03, and *Komarekiella* sp. 89.12. Biomass cultivation on BG11 compared to cultivation on nitrogen-depleted BG 11_0_, (two-sided *t*-test: *p < 0.05; **p < 0.01; n = 3).

*Nostoc* sp. 20.02, *Desmonostoc muscorum* 90.03, and *Komarekiella* sp. 89.12 produce enhanced amounts of EPS under nitrogen-limited cultivation conditions. Adsorption experiments with biomass produced under these conditions displayed a higher maximum adsorption capacity for REEs compared to biomass produced under standard conditions (Figure 3). A two-sided *t*-test revealed significant (*p* < 0.05) and highly significant (*p* < 0.01) differences in maximum adsorption capacity for REEs, especially for *Nostoc* sp. 20.02.

### 3.3 Maximum adsorption capacity of EPS, biomass after EPS extraction, and untreated biomass for REEs

**FIGURE 4:**
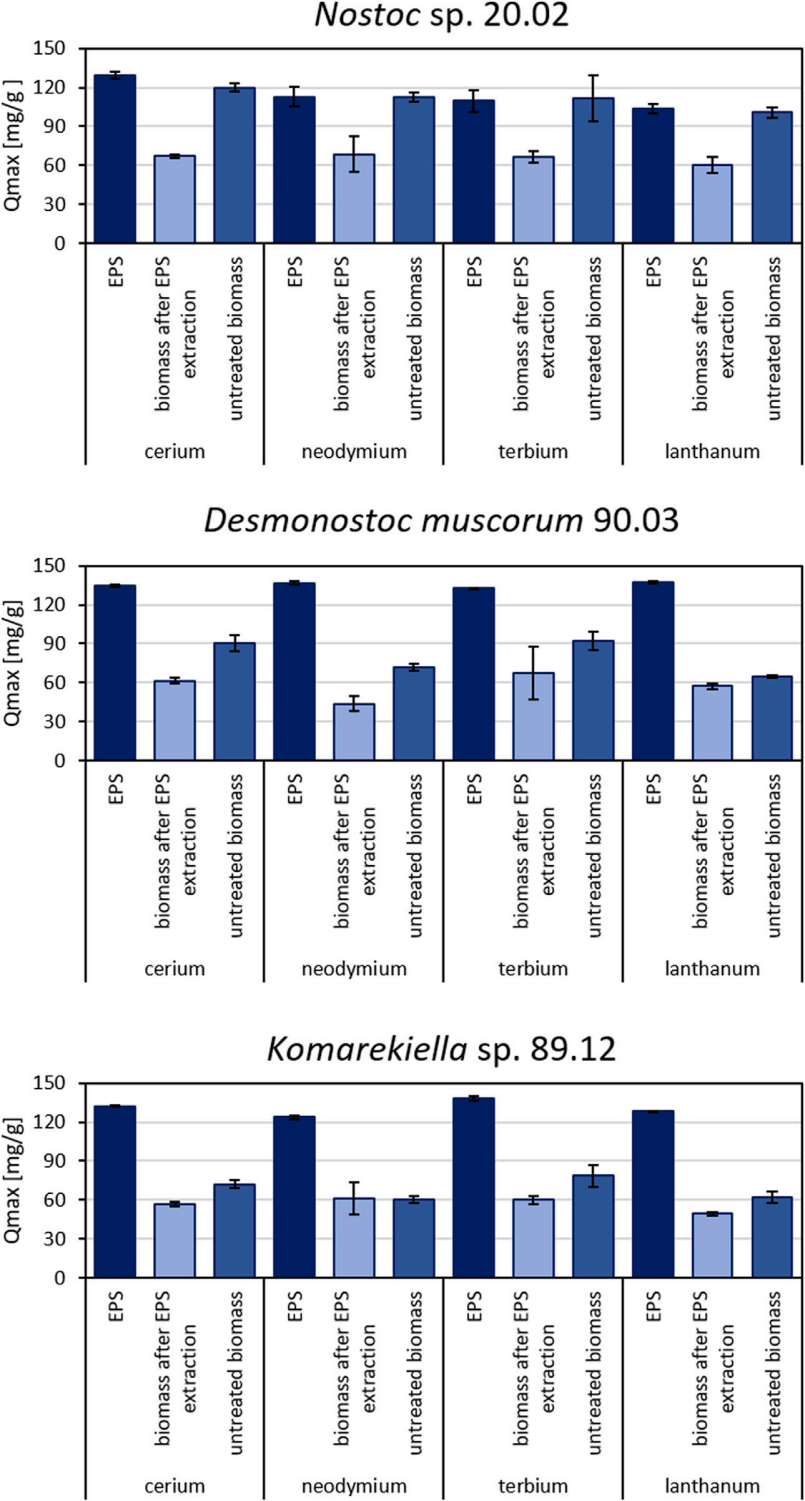
Maximum adsorption capacity for cerium, neodymium, terbium, and lanthanum for different biomass samples of *Nostoc* sp. 20.02, *Desmonostoc muscorum* 90.03, and *Komarekiella* sp. 89.12. Statistically significant differences in metal adsorption capacity were determined in a two-way ANOVA (*p* < 0.05; n = 3), followed by Tukey’s *post hoc* test. (For detailed significant differences see Supplementary Tables S2–S5).

Extracted EPS of all three tested cyanobacteria displayed high adsorption capacities for REEs (Figure 4). The separation of EPS from cyanobacterial biomasses significantly reduced the capacity for metal adsorption of the remaining biomass for all tested strains. Consequently, the maximum adsorption capacity of EPS for all tested metals was significantly higher than the maximum adsorption capacity of EPS-extracted biomass for all three tested cyanobacteria (see Supplementary Material). Although the differences were not as pronounced as for EPS samples, the metal adsorption capacities of EPS-extracted and untreated biomass varied significantly for *Desmonostoc muscorum* 90.03 and *Komarekiella* sp. 89.12. Regarding Ce, Nd, and La, *Nostoc* sp. 20.02 had the significantly highest adsorption capacity for all treatments except for extracted EPS. The extracted EPS of *Desmonostoc muscorum* 90.03 showed the most significantly highest adsorption capacity within the tested species. While differences between treatments exist, no significant differences in adsorption capacities between species exist for Tb. The adsorption of Nd by *Komarekiella* sp. 89.12 and Tb by *Desmonostoc muscorum* 90.03 were an exception in that context. Interestingly, untreated biomass and EPS from *Nostoc* sp. 20.02 displayed similar adsorption capacities for the tested REEs (see Supplementary Material).

### 3.4 FT-IR analysis of EPS before and after incubation in cerium(III) nitrate solution

**FIGURE 5:**
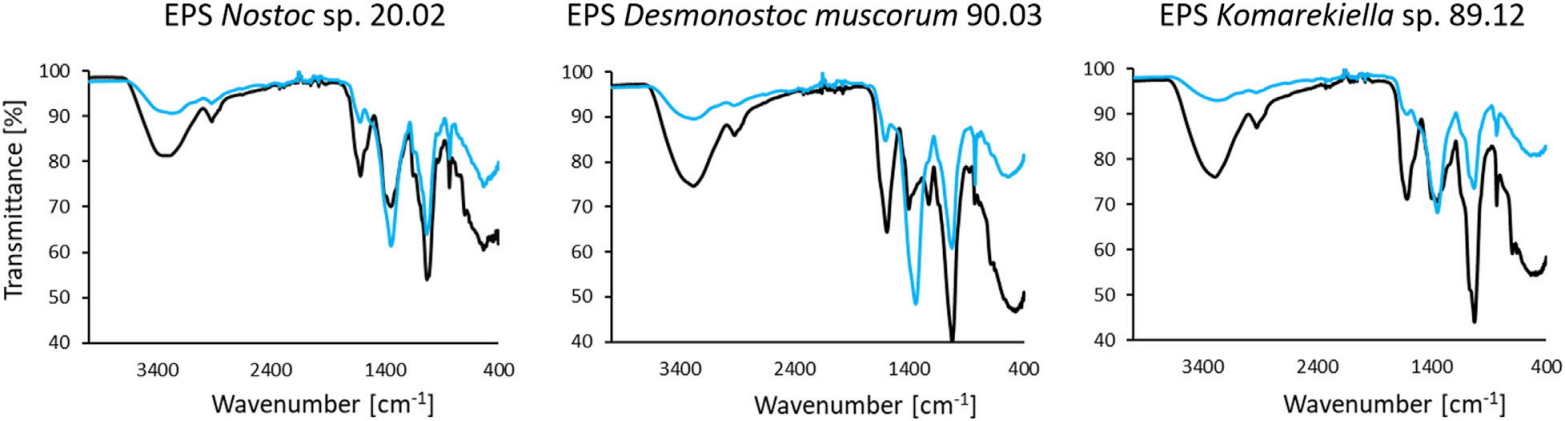
FT-IR spectra of EPS samples separated from indicated species’ biomass before (black) and after incubation in cerium(III) nitrate solution (1 μmol mg^−1^ biomass) (blue).

The IR spectra of all three analyzed EPS samples displayed signals that are characteristic of substances comprising polysaccharides (Figure 5). The broad band in the region between 3,500 and 3,200 cm^−1^ in the spectra can be assigned to the stretching vibrations of hydroxyl groups, whereas the signal at approximately 2,900 cm^−1^ is attributed to C-H stretching vibrations of CH_2_ groups (Liang and Marchessault, 1959). The presence of carboxyl groups is indicated by signals at 1,630 cm^-1^, which are linked to C=O stretching vibrations (Qian et al., 2009). Distinct signals at approximately 1,040 cm^-1^ can be assigned to C-O stretching vibration in polysaccharides (Nakamoto, 2009). Furthermore, the signals at approximately 1,340 cm^-1^ and in the area of 830 cm^-1^ could be attributed to asymmetric and symmetric stretching vibrations of NO_3_ (Trivedi et al., 2015). The interaction of biomass with the adsorbed metal ions induced changes in intensity and shifts in position for some signals in the FT-IR spectra following contact with cerium(III) nitrate.

Most notably, an attenuation of intensity is observed in the region between 3,500 and 3,200 cm^–1^ for all samples, indicating a decrease in free hydroxyl groups in the biomass (Mitic-Stojanovic et al., 2011). Likewise, direct interactions with carboxyl groups are indicated by differences in signal intensities at approximately 1,630 cm^-1^ and 1,040 cm^−1^ (Qian et al., 2009).

## 4 Discussion

Although it is accepted that EPS production is triggered under unfavorable metabolic conditions, such as N and/or P limitations (Flaibani et al., 1989; Vincenzini et al., 1990), this does not generally hold true. Nicolaus et al. (1999) reported a dramatic decrease in EPS production for two *Anabaena* species, which was also reported earlier for another *Anabaena* species byLama et al. (1996). This is in line with results by De Philippis et al. (1998) and Khattar et al. (2010) who found the same effect for five strains of *Cyanothece* and *Limnothrix redekei*, respectively. One reason might be that all of these strains are aquatic cyanobacteria, which rely less on the protecting properties of EPS during nutrient-limiting periods. The water body, e.g., alleviates temperature shifts and enhances the equal distribution of nutrients, and desiccation is rarely occurring—some of the primary protecting purposes of EPS. Alternatively, cyanobacteria can invest their photosynthetic carbon overflow during nutrient-limited conditions in the synthesis of intracellular energy storage compounds, such as glycogen, rather than releasing excess amounts of mucilage. For terrestrial cyanobacteria like the three strains examined in our study, an opposing situation is often reported: most of the cyanobacteria grow somehow attached to a substrate like soil or the surface of stones, where the contact between the substrate and the organisms is the site of nutrient uptake. These nutrients can only be taken up or distributed during humid periods when they are dissolved in the water layer. During these times, EPS play a crucial role as this is the contact interface between the cells and the substrate, e.g., the EPS of thalli formed by *Nostoc commune* can drastically increase their volume (Shaw et al., 2003; Tamaru et al., 2005), effectively absorbing water and nutrients. During subsequent desiccation periods, the EPS matrix can retain water and nutrients, which can be delivered to the cells, resulting in an extended phase of photosynthetic activity and, therewith, growth. In terrestrial cyanobacteria, the EPS consist of at least 10%–20% of dry matter (Huang et al., 1998), and under biotechnological control, the production can be induced to 50% of DM by drought stress at air-exposed cultivation (emersed) in biofilm-photobioreactors (Strieth et al., 2017; Lakatos and Strieth, 2018). In addition, a lot of terrestrial genera—and especially those of the Nostocales, such as *Nostoc*, *Desmonostoc*, and *Komarekiella*, that were part of this study—have complex life cycles (Figure 1) during which they undergo cell differentiation (Mollenhauer et al., 1994; Hrouzek et al., 2013; Johansen et al., 2017). These cyanobacteria not only have the ability to produce different types of cells, such as akinetes, which serve as resting stages that come along with a thick cell wall and EPS capsule (Figure 1E) but can also create cell stages, such as mobile hormogonia with a weakly pronounced EPS sheath (Figure 1A). Additionally, primordia and adult stages can be formed, which display extraordinarily thick EPS capsules surrounded by S-EPS (Mollenhauer et al., 1994; Hrouzek et al., 2013; Johansen et al., 2017). These differences are especially pronounced in terrestrial cyanobacteria compared to aquatic species and might indicate that terrestrial cyanobacterial strains have multiple triggers to metabolically invest in EPS during adverse environmental conditions. This opens new prospects for biosorption and other biotechnological approaches linked to cyanobacterial EPS because their formation and the properties of a strain, such as the produced EPS amounts, can be artificially triggered and directed (e.g., Moore et al., 2005; Sukenik et al., 2013). This is especially interesting for the strains tested here because they can grow under N-limitation due to the presence of heterocytes that bind N from the atmosphere. Our results demonstrate that the maximum adsorption capacity for Ce, Nd, Tb, and La was significantly increased for all strains cultivated under N-limited conditions compared to those grown under non-limiting growth conditions (Figure 3). This effect can be explained by i) a change in the composition of the EPS caused by the N-limitation, which consequently increases the adsorption capacity, or ii) an overproduction of EPS in response to N-limitation, resulting in a greater number of binding sites for REE biosorption. Although both effects can hardly be differentiated in this study, one can make the following conclusions: cultivation under N-limitation of the three tested strains significantly increased the maximum adsorption capacity for REE. This is in accordance with previous studies on metal adsorption by cyanobacterial EPS, where a direct positive correlation between metal uptake and EPS presence has been reported (Singh et al., 1998; Tien et al., 2005; Cui et al., 2021).

Interestingly, all extracted EPS samples exhibited comparable adsorption capacities for the tested REEs (with the exception of lanthanum, see Supplementary Material), whereas there were significant differences for untreated biomass. Similar adsorption capacities of EPS were observed despite variations in the monomeric EPS sugar composition. This might indicate an equally strong interaction of different sugar moieties with metal ions or the presence of other metal-interacting compounds in the EPS, such as proteins or lipids (Yadav et al., 2020). However, the content of proteins is mostly less than 10% in the EPS, and under N-limiting conditions—where higher adsorption capacities of separated EPS were found in this study—less proteins are produced arguing against an important role of proteins in REE adsorption, although special metal-binding proteins are synthesized by cyanobacteria such as metallothionein (Turner and Robinson, 1995). In this context, the analysis of FT-IR spectra indicated typical patterns for polysaccharide structures and indicated a dominant contribution of carbohydrate-associated hydroxyl and carboxyl groups during metal adsorption for all samples, which indicates a polysaccharide-driven mechanism. Metal adsorption by biological materials is often connected to the interaction of metal cations with hydroxyl and carboxyl groups (Ngah and Hanafiah, 2008; Javanbakht et al., 2014). EPS extracted from *Desmonostoc muscorum* 90.03 and *Komarekiella* sp. 89.12 comprised approximately 6%–10% glucuronic acids. Under standard environmental conditions, the carboxyl groups of uronic acids are partially ionized in aqueous solutions and contribute negative charges to the EPS polymers. These negative charges attract metal cations and support their sequestration.

In contrast, the EPS derived from *Nostoc* sp. 20.02 only contained small amounts of uronic acids. However, subsequent chemical hydrolysis released elevated amounts of acetic acid. The occurrence of acetic acid indicates the presence of acetylated sugar compounds in the EPS polymer. These building blocks can interact with metal ions in a same manner as uronic acids. This is in accordance with the FT-IR spectra, which indicated the involvement of carboxyl groups in metal adsorption for all strains tested in this study.

Compared to isolated EPS, the adsorption capacity for REE of biomass was significantly reduced if EPS were removed. Nevertheless, the EPS-extracted biomass still adsorbed metals from aqueous solutions. This indicates that REEs interact with components of the cyanobacterial cell wall. Compared to EPS, the cell wall might display fewer active binding sites or functional moieties and may exhibit a weaker REE affinity. For *Nostoc* sp. 20.02, the differences in metal adsorption capacity between EPS and untreated biomass were small. This suggests a major contribution of EPS to the overall metal adsorption in this cyanobacterium. Crude *Nostoc* sp. 20.02 biomass might have been fully encapsulated by EPS and, therefore, displayed similar adsorption properties to those of extracted EPS. On the other hand, differences in adsorption capacity between untreated biomass and EPS-extracted biomass for *Desmonostoc muscorum* 90.03 and *Komarekiella* sp. 89.12 indicate a supporting role of EPS in the total metal uptake.

Further investigations are required to determine the extent of the influence of EPS on the major mechanisms of overall adsorption. The differences between untreated and EPS-extracted biomass are mostly significant, but EPS-extracted biomass still had adsorption capacities of an average of 59.2% ± 2% (*Nostoc* sp. 20.02), 72.5% ± 12% (*Desmonostoc muscorum* 90.03), and 84.1% ± 11% (*Komarekiella* sp. 89.12) compared to untreated biomass. However, 16% (*Komarekiella* sp. 89.12)–41% (*Nostoc* sp. 20.02) of REE adsorption involves the extracellular passive binding of EPS (biosorption) caused by their chemical composition, especially that of polysaccharides. Moreover, separated EPS have even higher adsorption capacities than the intact biomass of the cyanobacteria assayed in this study.

In the context of a potential biosorption-based application for metal recovery, the continuous removal of EPS from a cyanobacterial culture during cultivation might be a feasible approach (Strieth et al., 2017). After metal adsorption, the separated EPS can be dried, and the resulting metal-enriched powder can be further processed for metal recovery. Cyanobacteria usually survive EPS extraction and can be reused for biomass and EPS production.

EPS extracted from cyanobacterial biomass demonstrated notable adsorption properties for REEs, suggesting their potential as an effective adsorbent for the separation and recovery of these valuable elements from wastewater or industrial effluents. The maximum adsorption capacity for REEs of the extracted EPS was higher than other adsorbents that have been investigated for metal recovery from aqueous solutions, including composite materials or untreated and modified biomass (see Table 1). Based on these promising results, further research and optimization are necessary to determine the feasibility and efficiency of EPS in an adsorption-based metal recovery process.

**TABLE 1:** Comparison of the maximum adsorption capacity for rare earth elements of various adsorbents.

Adsorbent	Metal studied	Q_max_ [mg/g]	Reference
*Calothrix brevissima* biomass and *Chlorella kessleri* biomass	Nd^3+^	67.8	Heilmann et al. (2021)
	Eu^3+^	50.1	
	Nd^3+^	53.4	
	Eu^3+^	16.7	
Nanocomposite of calcium alginate carrying poly (pyrimidine-thiophene-amide), CA-P(P-T-A)-NZFO	Nd^3+^	72.5	Javadian et al. (2020a)
	Tb^3+^	108.8	
	Dy^3+^	113.1	
Nanocomposite of calcium alginate/carboxymethyl chitosan, CA/CMC/Ni_0.2_Zn_0.2_Fe_2.6_O_4_	Nd^3+^	73.4	Javadian et al. (2020b)
	Tb^3+^	101.6	
	Dy^3+^	114.7	
Magnetic alginate–chitosan gel beads	La^3+^	97.1	Wu et al. (2011)
Chitosan–manganese–ferrite beads	Nd^3+^	44.3	Durán et al. (2020)
Alginate–lignin composite	Ce^3+^	98.0	Fila et al. (2022)
	Nd^3+^	98.0	
	Pr^3+^	98.7	
	La^3+^	109.6	
*Escherichia coli* biomass	Nd^3+^	30.9	Hosomomi et al. (2013)
	Dy^3+^	32.7	
	Lu^3+^	42.7	
Calcium-loaded *Sargassum polycystum* biomass	La^3+^	40.3	Diniz and Volesky (2005)
	Eu^3+^	62.3	
	Yb^3+^	48.4	
EPS of *Nostoc* sp. 20.02, EPS of *Desmonostoc muscorum* 90.03, and EPS of *Komarekiella* sp. 89.12	Ce^3+^	129.3 ± 2.7	This study
	Nd^3+^	112.9 ± 7.6	
	Tb^3+^	109.5 ± 8.2	
	La^3+^	103.5 ± 3.7	
	Ce^3+^	134.8 ± 0.7	
	Nd^3+^	136.7 ± 1.3	
	Tb^3+^	133.1 ± 0.2	
	La^3+^	137.4 ± 1.3	
	Ce^3+^	132.4 ± 0.5	
	Nd^3+^	123.9 ± 1.3	
	Tb^3+^	138.2 ± 1.9	
	La^3+^	128.5 ± 0.4	

For process development, important parameters and adsorption characteristics have to be examined, including adsorption kinetics, isotherms, and the influence of the pH value on metal uptake. Furthermore, adsorption–desorption cycles are commonly employed in the recovery of valuable materials, and the reversibility of the adsorption process is a key factor for environmentally friendly and cost-effective process implementation. Most important is probably the evaluation of additional and different cyanobacterial strains in terms of EPS and adsorption of REEs to determine the efficiency of the process and to further untangle strain-specific and general properties of the overall process. The desorption characteristics of REEs from EPS and biomass long-term stability have to be investigated to assess the viability of using EPS in practical applications.

## Data Availability

The original contributions presented in the study are included in the article/Supplementary Material; further inquiries can be directed to the corresponding author.
