# Supplementary material for: Stripped: contribution of cyanobacterial extracellular polymeric substances to the adsorption of rare earth elements from aqueous solutions
Source: Front Bioeng Biotechnol. 2023 Dec 20;11:1299349. doi: 10.3389/fbioe.2023.1299349 (PMC10762542; doi:10.3389/fbioe.2023.1299349)
Supplement: Supplementary file 1 [file Table1.DOCX]

Supplementary Material

# Supplementary Data

**Supplementary Table S1.** Sugar composition of isolated cyanobacterial EPS

| Sugars / organic acids | *Nostoc* sp. 20.02 | | *Desmonostoc muscorum* 90.03 | | *Komarekiella* sp*.* 89.12 | |
| --- | --- | --- | --- | --- | --- | --- |
|  | concentration [mg/g] | relative concentration [%] | concentration [mg/g] | relative concentration [%] | concentration [mg/g] | relative concentration [%] |
| Formic Acid | 11.4 ± 14.8 | 3.6 | 16.9 ± 15.8 | 4.7 | 1.1 ± 0.5 | 0.4 |
| Acetic acid | 32.4 ± 34.9 | 10.1 | 9.0 ± 7.8 | 2.5 | 6.2 ± 6.2 | 2.3 |
| Glucuronic acid | 2.6 ± 2.9 | 0.8 | 36.8 ± 13.8 | 10.3 | 16.7 ± 1.7 | 6.3 |
| Galacturonic acid | 1.1 ± 0.4 | 0.3 | 4.7 ± 2.6 | 1.3 | 1.1 ± 1.0 | 0.4 |
| Arabinose | n.d. | 0.0 | n.d. | 0.0 | 9.0 ± 0.7 | 3.4 |
| Fucose | 4.9 ± 5.4 | 1.5 | 16.7 ± 10.5 | 4.7 | 44.4 ± 2.1 | 16.9 |
| Glucose | 136.7 ± 3.22 | 42.7 | 54.0 ± 9.6 | 15.0 | 78.2 ± 1.2 | 29.8 |
| Mannitol | n.d. | 0.0 | 0.2 ± 0.3 | 0.1 | 0.2 ± 0.3 | 0.1 |
| Rhamnose | 63.9 ± 8.8 | 20.0 | 93.0 ± 23.2 | 26.0 | 17.3 ± 0.3 | 6.6 |
| Xylose, mannose,  galactose, fructose | 67.3 ± 4.8 | 21.0 | 127.6 ± 15.7 | 36.0 | 88.5 ± 1.3 | 33.7 |

Supplementary Table S2. (A) Two-way ANOVA tests (multivariate treatment conditions) of Cerium adsorption capacities depending on the species (*Nostoc* sp*.* 20.02 = Nost; *Desmonostoc muscorum* 90.03 = Desmo; *Komarekiella* sp. 89.12 = Koma) and treatments (Treat: separated EPS = EPS; EPS separated biomass = Bio-EPS; untreated biomass = Bio+EPS) and (B) checked for interactions by Tukey's post-hoc test (p < 0.05).

| FIXED-EFFECTS TWO-WAY ANOVA | | |  |  |  |
| --- | --- | --- | --- | --- | --- |
|  |  |  |  |  |  |
| **Cerium** | **Sum of sqrs** | **df** | **Mean square** | **F** | **p (same)** |
| Species: | 1.520 | 2 | 760,4 | 93,38 | 3,14E-10 |
| Treat: | 22.402 | 2 | 11.201 | 1375 | 2,07E-20 |
| Interaction: | 2.187 | 4 | 546,9 | 67,16 | 1,43E-10 |
| Within: | 146 | 18 | 8,143 |  |  |
| Total: | 26.257 | 26 |  |  |  |

| INTERACTION | |  |  |
| --- | --- | --- | --- |
| **Cerium** |  | **Q** | **p** |
| Nost-EPS | Nost-Bio-EPS | 37,79 | 3,95E-14 |
| Nost-EPS | Nost-Bio+EPS | 5,577 | 0,01364 |
| Nost-EPS | Koma-EPS | 1,867 | 0,834 |
| Nost-EPS | Desmo-EPS | 3,344 | 0,2684 |
| Nost-Bio-EPS | Nost-Bio+EPS | 32,21 | 2,64E-13 |
| Nost-Bio-EPS | Koma-Bio-EPS | 6,248 | 0,005048 |
| Nost-Bio-EPS | Desmo-Bio-EPS | 3,516 | 0,2213 |
| Nost-Bio+EPS | Koma-Bio+EPS | 29,05 | 1,15E-12 |
| Nost-Bio+EPS | Desmo-Bio+EPS | 18,04 | 3,50E-09 |
| Koma-EPS | Koma-Bio-EPS | 45,91 | 2,55E-14 |
| Koma-EPS | Koma-Bio+EPS | 36,49 | 5,37E-14 |
| Koma-EPS | Desmo-EPS | 1,477 | 0,9363 |
| Koma-Bio-EPS | Koma-Bio+EPS | 9,415 | 5,25E-05 |
| Koma-Bio-EPS | Desmo-Bio-EPS | 2,732 | 0,4864 |
| Koma-Bio+EPS | Desmo-Bio+EPS | 11,01 | 6,45E-06 |
| Desmo-EPS | Desmo-Bio-EPS | 44,65 | 2,55E-14 |
| Desmo-EPS | Desmo-Bio+EPS | 26,96 | 3,75E-12 |
| Desmo-Bio-EPS | Desmo-Bio+EPS | 17,69 | 4,80E-09 |

Supplementary Table S3. (A) Two-way ANOVA tests (multivariate treatment conditions) of Neodymium adsorption capacities depending on the species (*Nostoc* sp. 20.02 = Nost; *Desmonostoc muscorum* 90.03 = Desmo; *Komarekiella* sp. 89.12 = Koma) and treatments (Treat: separated EPS = EPS; EPS separated biomass = Bio-EPS; untreated biomass = Bio+EPS) and (B) checked for interactions by Tukey's post-hoc test (p < 0.05).

| FIXED-EFFECTS TWO-WAY ANOVA | | |  |  |  |
| --- | --- | --- | --- | --- | --- |
|  |  |  |  |  |  |
| **Neodymium** | **Sum of sqrs** | **df** | **Mean square** | **F** | **p (same)** |
| Species: | 1372,8 | 2 | 686,402 | 13,18 | 0,0002978 |
| Treat: | 20492,2 | 2 | 10246,1 | 196,8 | 5,85E-13 |
| Interaction: | 4947,34 | 4 | 1236,83 | 23,76 | 5,65E-07 |
| Within: | 937,166 | 18 | 52,0648 |  |  |
| Total: | 27749,5 | 26 |  |  |  |

| INTERACTION |  |  |  |
| --- | --- | --- | --- |
| **Neodymium** |  | **Q** | **p** |
| Nost-EPS | Nost-Bio-EPS | 10,63 | 1,04E-05 |
| Nost-EPS | Nost-Bio+EPS | 0,0947 | 1 |
| Nost-EPS | Koma-EPS | 2,63 | 0,5284 |
| Nost-EPS | Desmo-EPS | 5,712 | 0,01118 |
| Nost-Bio-EPS | Nost-Bio+EPS | 10,54 | 1,18E-05 |
| Nost-Bio-EPS | Koma-Bio-EPS | 1,744 | 0,8721 |
| Nost-Bio-EPS | Desmo-Bio-EPS | 5,954 | 0,007811 |
| Nost-Bio+EPS | Koma-Bio+EPS | 12,51 | 1,04E-06 |
| Nost-Bio+EPS | Desmo-Bio+EPS | 9,736 | 3,40E-05 |
| Koma-EPS | Koma-Bio-EPS | 15,01 | 6,57E-08 |
| Koma-EPS | Koma-Bio+EPS | 15,23 | 5,20E-08 |
| Koma-EPS | Desmo-EPS | 3,082 | 0,3526 |
| Koma-Bio-EPS | Koma-Bio+EPS | 0,2259 | 1 |
| Koma-Bio-EPS | Desmo-Bio-EPS | 4,21 | 0,09372 |
| Koma-Bio+EPS | Desmo-Bio+EPS | 2,772 | 0,47 |
| Desmo-EPS | Desmo-Bio-EPS | 22,3 | 1,06E-10 |
| Desmo-EPS | Desmo-Bio+EPS | 15,54 | 3,79E-08 |
| Desmo-Bio-EPS | Desmo-Bio+EPS | 6,756 | 0,002372 |

Supplementary Table S4. (A) Two-way ANOVA tests (multivariate treatment conditions) of Terbium adsorption capacities depending on the species (*Nostoc* sp. 20.02 = Nost; *Desmonostoc muscorum* 90.03 = Desmo; *Komarekiella* sp. 89.12 = Koma) and treatments (Treat: separated EPS = EPS; EPS separated biomass = Bio-EPS; untreated biomass = Bio+EPS) and (B) checked for interactions by Tukey's post-hoc test (p < 0.05).

| FIXED-EFFECTS TWO-WAY ANOVA | | |  |  |  |
| --- | --- | --- | --- | --- | --- |
|  |  |  |  |  |  |
| **Terbium** | **Sum of sqrs** | **df** | **Mean square** | **F** | **p (same)** |
| Species: | 131,639 | 2 | 65,8195 | 0,6279 | 0,545 |
| Treat: | 17529,4 | 2 | 8764,7 | 83,62 | 7,73E-10 |
| Interaction: | 2993,16 | 4 | 748,291 | 7,139 | 0,001259 |
| Within: | 1886,73 | 18 | 104,818 |  |  |
| Total: | 22540,9 | 26 |  |  |  |

| INTERACTION |  |  |  |
| --- | --- | --- | --- |
| **Terbium** |  | **Q** | **p** |
| Nost-EPS | Nost-Bio-EPS | 7,277 | 0,001099 |
| Nost-EPS | Nost-Bio+EPS | 0,3398 | 1 |
| Nost-EPS | Koma-EPS | 4,847 | 0,03918 |
| Nost-EPS | Desmo-EPS | 3,992 | 0,1243 |
| Nost-Bio-EPS | Nost-Bio+EPS | 7,617 | 0,0006677 |
| Nost-Bio-EPS | Koma-Bio-EPS | 1,108 | 0,9837 |
| Nost-Bio-EPS | Desmo-Bio-EPS | 0,1135 | 1 |
| Nost-Bio+EPS | Koma-Bio+EPS | 5,546 | 0,01427 |
| Nost-Bio+EPS | Desmo-Bio+EPS | 3,22 | 0,3062 |
| Koma-EPS | Koma-Bio-EPS | 13,23 | 4,49E-07 |
| Koma-EPS | Koma-Bio+EPS | 10,05 | 2,22E-05 |
| Koma-EPS | Desmo-EPS | 0,8549 | 0,9958 |
| Koma-Bio-EPS | Koma-Bio+EPS | 3,179 | 0,3195 |
| Koma-Bio-EPS | Desmo-Bio-EPS | 1,222 | 0,9736 |
| Koma-Bio+EPS | Desmo-Bio+EPS | 2,326 | 0,6577 |
| Desmo-EPS | Desmo-Bio-EPS | 11,16 | 5,36E-06 |
| Desmo-EPS | Desmo-Bio+EPS | 6,873 | 0,001996 |
| Desmo-Bio-EPS | Desmo-Bio+EPS | 4,283 | 0,08512 |

Supplementary Table S5. (A) Two-way ANOVA tests (multivariate treatment conditions) of Lanthanum adsorption capacities depending on the species (*Nostoc* sp. 20.02 = Nost; *Desmonostoc muscorum* 90.03 = Desmo; *Komarekiella* sp. 89.12 = Koma) and treatments (Treat: separated EPS = EPS; EPS separated biomass = Bio-EPS; untreated biomass = Bio+EPS) and (B) checked for interactions by Tukey's post-hoc test (p < 0.05).

| FIXED-EFFECTS TWO-WAY ANOVA | | |  |  |  |
| --- | --- | --- | --- | --- | --- |
|  |  |  |  |  |  |
| **lanthanum** | Sum of sqrs | df | Mean square | F | p (same) |
| Species: | 344,852 | 2 | 172,426 | 15,92 | 0,0001047 |
| Treat: | 21542 | 2 | 10771 | 994,2 | 3,76E-19 |
| Interaction: | 4513,77 | 4 | 1128,44 | 104,2 | 3,45E-12 |
| Within: | 195,008 | 18 | 10,8338 |  |  |
| Total: | 26595,6 | 26 |  |  |  |

| INTERACTION |  |  |  |
| --- | --- | --- | --- |
| **lanthanum** |  | Q | p |
| Nost-EPS | Nost-Bio-EPS | 22,59 | 8,47E-11 |
| Nost-EPS | Nost-Bio+EPS | 1,516 | 0,9286 |
| Nost-EPS | Koma-EPS | 13,14 | 4,97E-07 |
| Nost-EPS | Desmo-EPS | 17,86 | 4,13E-09 |
| Nost-Bio-EPS | Nost-Bio+EPS | 21,08 | 2,76E-10 |
| Nost-Bio-EPS | Koma-Bio-EPS | 5,826 | 0,009447 |
| Nost-Bio-EPS | Desmo-Bio-EPS | 1,69 | 0,8872 |
| Nost-Bio+EPS | Koma-Bio+EPS | 20,43 | 4,64E-10 |
| Nost-Bio+EPS | Desmo-Bio+EPS | 18,93 | 1,61E-09 |
| Koma-EPS | Koma-Bio-EPS | 41,56 | 2,71E-14 |
| Koma-EPS | Koma-Bio+EPS | 35,09 | 8,39E-14 |
| Koma-EPS | Desmo-EPS | 4,712 | 0,04738 |
| Koma-Bio-EPS | Koma-Bio+EPS | 6,479 | 0,003582 |
| Koma-Bio-EPS | Desmo-Bio-EPS | 4,136 | 0,1033 |
| Koma-Bio+EPS | Desmo-Bio+EPS | 1,499 | 0,9319 |
| Desmo-EPS | Desmo-Bio-EPS | 42,14 | 2,67E-14 |
| Desmo-EPS | Desmo-Bio+EPS | 38,3 | 3,62E-14 |
| Desmo-Bio-EPS | Desmo-Bio+EPS | 3,842 | 0,15 |
